# The augmented value of using clinical notes in semi-automated surveillance of deep surgical site infections after colorectal surgery

**DOI:** 10.1186/s13756-023-01316-x

**Published:** 2023-10-26

**Authors:** Janneke D.M. Verberk, Suzanne D. van der Werff, Rebecka Weegar, Aron Henriksson, Milan C. Richir, Christian Buchli, Maaike S.M. van Mourik, Pontus Nauclér

**Affiliations:** 1 Department of Medical Microbiology and Infection Prevention, University Medical Centre Utrecht, Utrecht, the Netherlands; 2 Julius Centre for Health Sciences and Primary Care, University Medical Centre Utrecht, Utrecht, the Netherlands; 3 Department of Epidemiology and Surveillance, Centre for Infectious Diseases Control, National Institute for Public Health and the Environment, Bilthoven, the Netherlands; 4 Department of Medicine Solna, Division of Infectious Diseases, Karolinska Institutet, Stockholm, Sweden; 5 Department of Infectious Diseases, Karolinska University Hospital, Stockholm, Sweden; 6 Department of Computer and Systems Sciences, Stockholm University, Stockholm, Sweden; 7 Department of Surgery, Cancer Centre, University Medical Centre Utrecht, Utrecht, the Netherlands; 8 Department of Molecular Medicine and Surgery, Karolinska Institutet, Stockholm, Sweden; 9 Department of Pelvic Cancer, GI Oncology and Colorectal Surgery Unit, Karolinska University Hospital, Stockholm, Sweden

**Keywords:** Automated surveillance, Algorithm, Colorectal surgery, Healthcare-associated infections, Natural language processing, Surgical site infections

## Abstract

**Background:**

In patients who underwent colorectal surgery, an existing semi-automated surveillance algorithm based on structured data achieves high sensitivity in detecting deep surgical site infections (SSI), however, generates a significant number of false positives. The inclusion of unstructured, clinical narratives to the algorithm may decrease the number of patients requiring manual chart review. The aim of this study was to investigate the performance of this semi-automated surveillance algorithm augmented with a natural language processing (NLP) component to improve positive predictive value (PPV) and thus workload reduction (WR).

**Methods:**

Retrospective, observational cohort study in patients who underwent colorectal surgery from January 1, 2015, through September 30, 2020. NLP was used to detect keyword counts in clinical notes. Several NLP-algorithms were developed with different count input types and classifiers, and added as component to the original semi-automated algorithm. Traditional manual surveillance was compared with the NLP-augmented surveillance algorithms and sensitivity, specificity, PPV and WR were calculated.

**Results:**

From the NLP-augmented models, the decision tree models with discretized counts or binary counts had the best performance (sensitivity 95.1% (95%CI 83.5–99.4%), WR 60.9%) and improved PPV and WR by only 2.6% and 3.6%, respectively, compared to the original algorithm.

**Conclusions:**

The addition of an NLP component to the existing algorithm had modest effect on WR (decrease of 1.4–12.5%), at the cost of sensitivity. For future implementation it will be a trade-off between optimal case-finding techniques versus practical considerations such as acceptability and availability of resources.

## Background

Approximately 5–30% of colorectal surgery patients develop a surgical site infection (SSI). SSIs result in morbidity, mortality, longer hospital stays and extra costs [1–3]. Monitoring SSIs is an essential policy strategy and has been proven effective in reducing these infections [4, 5]. Several (local and national) surveillance programs target SSI after colorectal surgery; patient records are retrospectively reviewed and manually annotated by infection control practitioners (ICPs) according to surveillance case definitions for SSI [6–8]. This traditional way of performing surveillance is labour-intensive, prone to subjective interpretation, and poor interrater agreement has been reported [9–11]. In the past years, automated surveillance methods that re-use data stored in electronic health records (EHRs) are increasingly developed to reduce workload, and to objectify and align surveillance methods. They are considered an attractive alternative to manual surveillance [12].

For most automated surveillance algorithms targeting SSI after colorectal surgery, no satisfying results have been reported so far as the methods described are not applicable to different settings, are very complex, and have insufficient performance [13–17]. One semi-automated algorithm has been described and validated in multiple (Dutch) hospitals with promising results [18]. With the use of structured data from radiology orders, admission-and discharge dates, antibiotic prescriptions, and re-operations, the algorithm classifies patients into high-or low probability of having had a deep SSI according to pre-specified rules. Only the high-probability records need manual confirmation [19]. Despite high sensitivity, the workload reduction achieved was not optimal given the large number of false positives.

As the diagnosis of SSI is mainly dependent on physical examinations and observations that are described in clinical notes, the inclusion of unstructured, free-text information to this algorithm may improve the accuracy by reducing the number of false positives. Natural language processing (NLP) is a technique that processes, learns and understands human language content, and can be used in analysing these unstructured data [20]. Experiences with NLP-supported surveillance algorithms are limited and they provide varying and often inconclusive results [21–24]. Also, the combination of using both structured and unstructured data for surveillance algorithms has not been extensively researched so far, but might result in better performance and case-finding [25, 26]. The aim of this study was to investigate the performance of the original semi-automated surveillance algorithm augmented with an NLP component to improve positive predictive value (PPV) and to reduce the workload.

## Methods

### Study design, setting and study population

**Fig. 1:**
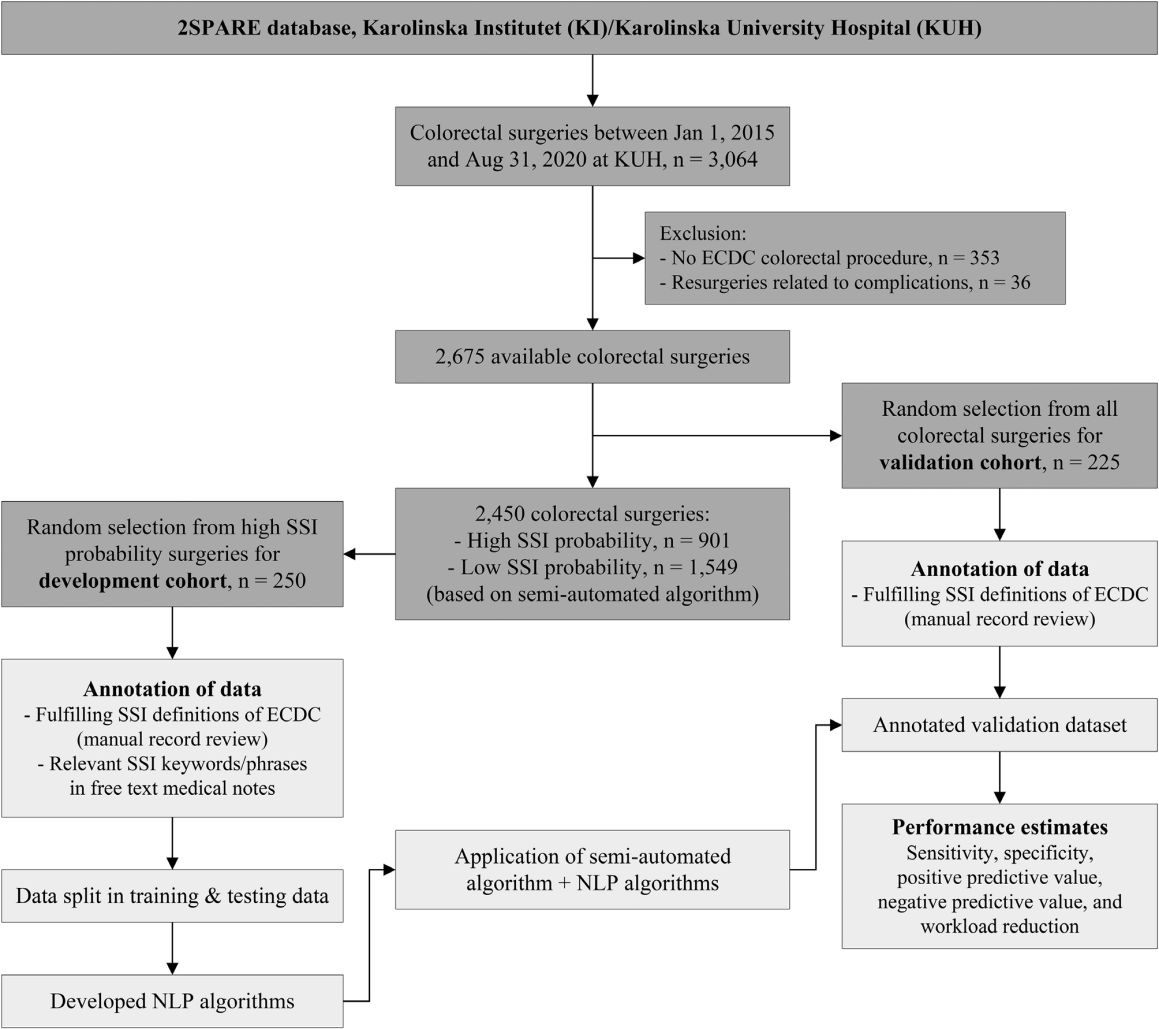
Flow chart of the study *ECDC: European Centre for Disease Prevention and Control; NLP: natural language processing; SSI: surgical site infection.* *Semi-automated algorithm as published in Verberk et al.* [18].

This is a retrospective, observational cohort study including patients undergoing colorectal surgery (i.e., primary or secondary colorectal resections, incisions or anastomosis) performed at the Karolinska University Hospital (KUH) Sweden, between January 1, 2015 and August 31, 2020. KUH is a tertiary care academic centre with 1,100 beds divided between two hospitals (Huddinge and Solna), which serves the population of Region Stockholm (2.3 million inhabitants). The original semi-automated algorithm [18] – based on structured data – was validated in KUH [27]. The same 225 randomly selected surgeries were also used as validation cohort for this study. The semi-automated algorithm was subsequently applied to the remaining colorectal surgeries and a random sample of 250 high-probability records were selected for the development cohort (Fig. 1). Model results were compared with the reference standard, which is the traditional manually annotated surveillance. The study was approved by the Regional Ethical Review Board in Stockholm, Sweden (2018/1030-31).

### Outcome

The outcome was deep SSI or organ/space SSI, hereinafter together referred to as deep SSI, versus no deep SSI within 30 days after the colorectal procedure. The outcome was recorded during manual annotation by two experienced ICPs according to the European Centre for Disease Prevention and Control (ECDC) SSI definition and guidelines at the time of this study [6].

### Data sources

The 2SPARE (2020 started Stockholm/Sweden Proactive Adverse Events REsearch) database is an SQL-based relational database and a duplicate of prospectively entered data from the EHR system of KUH, containing data on patient characteristics, hospital admission and discharge records, outpatient records, physiological parameters, medication, microbiology, clinical chemistry, radiology, and clinical notes. The clinical notes data includes unstructured, free-text notes such as progress notes, discharge summaries, history and physical examination notes, and telephone encounter notes, all written in the Swedish language. We limited the notes to those written by physicians, residents, surgery assistants, and nurses, and to those written within 1–30 days post-surgery, as these are most likely to contain SSI-relevant information.

### Development and validation of NLP-augmented algorithms

**Fig. 2:**
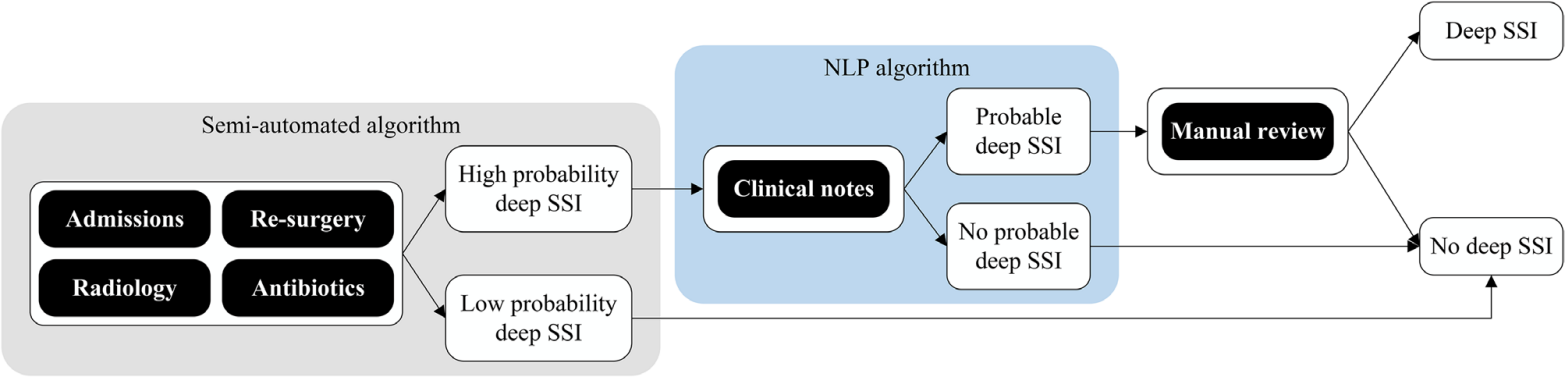
Flow diagram of natural language processing-augmented surveillance algorithm for deep surgical site infections *NLP: natural language processing; SSI: surgical site infection.* *Schematic overview of the original semi-automated algorithm comprised of structured data (grey frame), augmented with unstructured data from clinical notes (blue frame).* *Admissions: Length of stay of index admission ≥ 14 days or 1 readmission to original department or in-hospital mortality within follow-up (FU) time (= 45 days after surgery).* *Re-surgery: ≥1 reoperation by original surgery specialty after the index surgery but within FU time.* *Radiology: ≥1 orders for CT scan within FU time.* *Antibiotics: ≥3 consecutive days of antibiotics (ATC J01) within FU time, starting from day 2 (index surgery = day 0).* *Fulfilling 2–4 of the above components classifies surgery as high probability for deep SSI.*

The NLP algorithms were developed as an ‘add-on’ component and designed as an additional step following the existing semi-automated algorithm, aiming to reduce false-positive results whilst maintaining high sensitivity. This sequential design will arguably lower implementation thresholds in the future as hospitals can already start implementing the semi-automated algorithm with structured data and may later add the (more advanced and challenging) NLP component (Fig. 2). Several NLP components were developed using the development cohort consisting of high-probability individuals. The final NLP-augmented algorithms were validated using the validation cohort as described above (Fig. 1).

### Pre-processing of linguistic variables

A list of keywords was compiled by reviewing clinical literature and local case reports, and by expert consultation in the Netherlands and Sweden (i.e., colorectal surgeons, medical microbiologists, ICPs, infectious disease consultants). Next, from the keywords we created a list of lemmatized versions and applied part-of-speech tagging to capture differences in grammatical and spelling versions of the words. This resulted in the overall lexicon list. The keywords given by Dutch experts were translated to Swedish to be able to apply them on Swedish notes, and all keywords were translated to English for the purpose of reporting results.

### Feature selection and algorithm development

The original keywords and their lemmatized versions can be considered as ‘features’. All text from the clinical notes were matched with the lexicon list and each feature match was counted. Negation detection using the NegEx algorithm was applied to filter out negated mentions [28]. For example, in case ‘*no signs of infection*’ is written down, the keyword ‘*infection’* is negated and not counted as a keyword match. Subsequently, three input types were considered: a count per keyword, a discretized count with four bins, and a binary model indicating the presence or absence of a keyword. Each input type has its pros and cons: a binary representation benefits from its simplicity, however, cannot capture the case when several mentions of the same keyword corresponds to a stronger deep SSI signal. The count per keyword, on the other hand, captures the number of times each keyword is mentioned, but is more sensitive to writing styles and will have fewer examples of each distinct keyword count in the training data. The discretized count can be viewed as a compromise between the binary model and the counts model, since three of the bins represented a count below, within, or above the expected interquartile range, and the fourth bin represented no occurrences.

During development, we split the development cohort consisting of high-probability records as classified by the original algorithm into training (80%) and testing data sets (20%) to evaluate parameters of the learning algorithms. Two tree-based classification algorithms, a single decision tree (DT) and a random forest (RF) with 500 trees [29], were evaluated for their ability to separate between the two classes, deep SSI and no deep SSI [30, 31]. A DT has the benefit of being interpretable, since the tree can be understood as one set of rules for classifying future patients as belonging to either class. An RF, on the other hand, is a more complex model with multiple sets of rules and therefore lacks interpretability, but often outperforms a DT. Each of the classifiers, DT and RF, was applied to each feature representation (raw counts, discretized counts, or binary counts) resulting in six tree-based models.

For application in semi-automated surveillance, a near-perfect sensitivity is required as false-positive cases are corrected during subsequent chart review, whereas false-negative cases will remain unnoticed. To increase the sensitivity when using the DT classifier, ten small decision trees with slightly different characteristics were inferred from the development data. Subsequently, in the validation cohort, a patient was classified as deep SSI if any of these trees classified the patient as such. This ensemble of DT classifiers could be considered as a miniature forest with a decision threshold of 0.1. Within an RF, each tree classifies each patient in the data set. Generally, for an RF with two classes, a majority decision determined class membership, i.e., the class assigned by a majority of the trees will be assigned to the patient. This corresponds to a decision threshold of 0.5, meaning that 50% of the trees are required to consider a patient as belonging to the class deep SSI for the RF to classify it as such. To increase the sensitivity of the RF the conventional decision threshold of 0.5 was lowered, meaning that fewer trees are required to classify a patient as deep SSI, which will however reduce PPV. Multiple decision thresholds were explored using the development cohort, and the thresholds of 0.3 (for model using raw or discretized counts) and 0.35 (model using binary counts) were selected to ensure a high sensitivity (> 0.95 in the development cohort).

### Rule-based NLP component

**Fig. 3:**
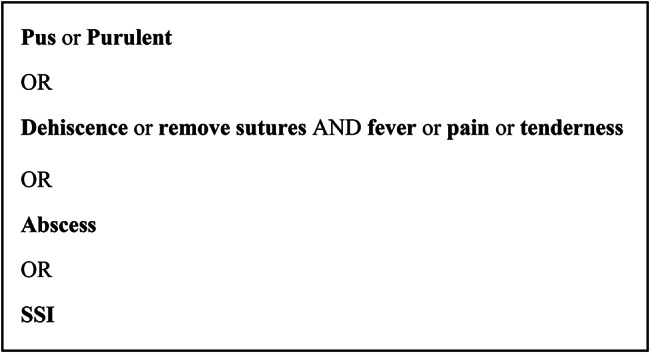
Rule-based component

Furthermore, a rule-based NLP component was developed based on keywords reflecting the deep SSI definition (Fig. 3). This NLP component is more straightforward as no DT or RF techniques are used, i.e., if a keyword match was present for a patient according to the OR/AND-rules as specified in the supplementary file, the patient was classified as probable deep SSI by the algorithm.

### Analysis

Baseline characteristics were compared between the high probability groups – as defined by the original algorithm – of the development and validation cohorts. Heat maps were created from the development cohort to visualize the presence of keywords between the deep SSI group and the group without. In total, seven surveillance models were compared, as described above, with the original semi-automated model composed of structured data only (model 1): model 1 augmented with the NLP component developed with DT using either raw counts (model 2), discretized counts (model 3) or binary counts (model 4); model 1 augmented with the NLP component developed with RF using either raw counts (model 5), discretized counts (model 6) or binary counts (model 7); and model 1 augmented with the rule-based component (model 8). The performance measures sensitivity (recall), specificity, PPV (precision), negative predictive value (NPV) and workload reduction (WR) with corresponding 95%-confidence intervals (95%CI) were calculated as compared to the reference standard. WR was defined as the difference between the total number of surgeries under surveillance and the proportion of surgeries requiring manual review after algorithm application. 2SPARE data acquisition, management and analysis were performed using R statistical software (version 3.6.1) and Python (version 3.7), and in accordance with current regulations concerning privacy and ethics.

## Results

The median age of the validation cohort was 66 year (IQR 55–75) and 48.9% (n = 110) were female (Table 1). The majority of patients had a primary surgery (63.6%, n = 143) and most surgeries were open (77.3%, n = 174). In 41.8% (n = 94) of patients a stoma was created. Baseline characteristics between the high probability deep SSI cases of the development cohort (n = 250) and the validation cohort (n = 96) were similar, albeit somewhat higher age, lower ASA classification, lower frequency of primary procedures, and higher rate of SSIs in the validation cohort (Table 1).

**Table 1:** Baseline characteristics of surgeries in validation and development cohort

	Validation cohort				Development cohort		*P* ^*#*^
	Total		High probability SSI		High probability SSI		
n	225		96		250		
Age (years), median [IQR]	66	[55–75]	68	[58–75]	65	[52–73]	0.088
Female sex, n (%)	110	(48.9)	37	(38.5)	92	(36.8)	0.860
BMI, median [IQR]	25.6	[22.3–29.4]	25.7	[22.3–29.7]	25.2	[22.6–28.6]	0.782
- Missing, n (%)	2	(0.9)	1	(1.0)	0	(0.0)	
ASA classification, n (%)							0.232
- Grade I	25	(11.1)	9	(9.4)	17	(6.8)	
- Grade II	96	(42.7)	35	(36.5)	125	(50.0)	
- Grade III	78	(34.7)	42	(43.8)	83	(33.2)	
- Grade IV	4	(1.8)	2	(2.1)	4	(1.6)	
- Grade V	0	(0)	0	(0)	0	(0)	
- Unknown	22	(9.8)	8	(8.3)	21	(8.4)	
Surgical approach, n (%)							0.583
- Closed	51	(22.7)	12	(12.5)	37	(14.8)	
- Open	174	(77.3)	84	(87.5)	213	(85.2)	
Duration of surgery (minutes), median [IQR]	316	[206–427]	371	[245–458]	379	[266–514]	0.372
- Missing, n (%)	65	(28.9)	21	(21.9)	70	(28.0)	
Wound contamination class, n (%)							0.771
- Clean-contaminated (class 2)	171	(76.0)	74	(77.1)	184	(73.6)	
- Contaminated (class 3)	42	(18.7)	16	(16.7)	50	(20.0)	
- Dirty-infected (class 4)	12	(5.3)	6	(6.3)	16	(6.4)	
Stoma, n (%)	94	(41.8)	54	(56.3)	139	(55.6)	1.000
Malignancy, n (%)	173	(76.9)	77	(80.2)	191	(76.4)	0.538
Primary procedure, n (%)	143	(63.6)	53	(55.2)	162	(64.8)	0.128
Anastomotic leakage, n (%) *	13	(31.7)	13	(32.5)	36	(39.1)	0.597
Surgical site infection, n (%)							0.298
- No	165	(73.3)	44	(45.8)	132	(52.8)	
- Yes	60	(26.7)	52	(54.2)	118	(46.4)	
- Superficial	19	(31.7)	12	(23.1)	26	(22.0)	
- Deep or organ/space	41	(68.3)	40	(76.9)	92	(78.0)	
30-day mortality, n (%)	5	(2.2)	3	(3.1)	6	(2.4)	0.998

*BMI: body mass index; ASA: Physical status classification developed by the American Society of Anesthesiologists*

* Only registered in case of deep SSI.

*# Comparison between development cohort and high probability deep SSI cases of validation cohort*

### Model performance of the semi-automated algorithm augmented with an NLP component

**Fig. 4:**
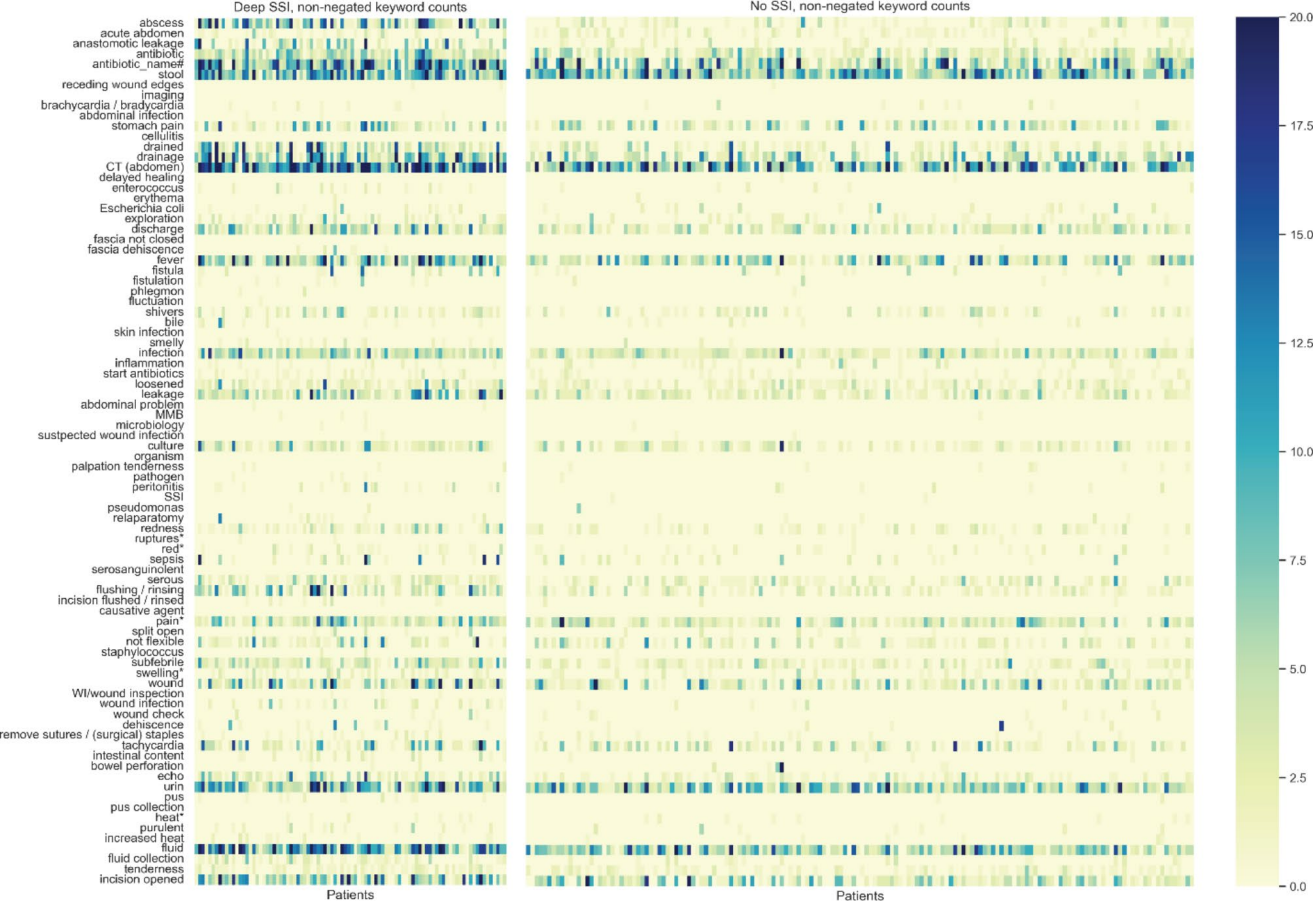
Heat map for the distribution of keywords among patients with and without deep SSI * Proximity search, keyword must be within a distance of five words from one of the following locations: incision, operation wound, abdomen, wound, pelvis, duodenum, flank, gall bladder, skin, intra-abdominal, next to anastomosis, colon, liver, pancreas, abdomen/stomach, between small intestines, operation area, operation wound, presacral, rectum, retroperitoneal, incision, intestine, small intestine, under diaphragm, midline incision, midline wound, sutures / stitches / (surgical) staples, stomach. *# The following antibiotics including their brand names: piperacillin-tazobactam, meropenem, imipenem, metronidazole, ciprofloxacin, cefotaxime, trimethoprim-sulfa, cefuroxime, amoxicillin.*

The distribution of keywords among patients with deep SSI versus no deep SSI differed with regards to frequency and timing (Fig. 4). The keywords ‘abscess’, ‘anastomotic leakage’, ‘drainage’ and antibiotic names appeared more frequently in the clinical notes from deep SSI cases. The other keywords were present in both groups, although more often in the group of patients with deep SSI and between day 15–30 post-surgery.

For each NLP-augmented surveillance model, performances are shown in Table 2. Model 3 and 4 had sensitivity above 95% and 3.6% less records to review manually as compared to the original algorithm based of structured data (model 1). Keywords incorporated in model 3 were: the antibiotic names, ‘abscess’, ‘anastomotic leakage’, ‘subfebrile’, ‘fluid’, ‘intestinal content’, ‘drainage’, ‘leakage’, ‘antibiotics’, and ‘drained’. For model 4 also the following keywords were included: ‘intestinal content’, ‘serous’, and ‘echo’. Model 8, with the rule-based component, had lowest sensitivity. Overall, the models with discretized or binary count input types had better performance estimates than the models with raw counts.

**Table 2:** Performance characteristics of the different surveillance models

	Sensitivity, % (95%CI)	Specificity, % (95%CI)	PPV, % (95%CI)	NPV, % (95%CI)	WR, %
Model 1	97.6 (87.1–100.0)	69.6 (62.4–76.1)	41.7 (31.7–52.2)	99.2 (95.7–100.0)	57.3
Model 2	87.8 (73.8–95.9)	79.9 (73.4–85.4)	49.3 (37.4–61.3)	96.7 (92.5–98.9)	67.5
Model 3	95.1 (83.5–99.4)	73.4 (66.4–79.6)	44.3 (33.7–55.3)	98.5 (94.8–99.8)	60.9
Model 4	95.1 (83.5–99.4)	73.4 (66.4–79.6)	44.3 (33.7–55.3)	98.5 (94.8–99.8)	60.9
Model 5	92.7 (80.0–98.5)	77.7 (71.0–83.5)	48.1 (36.7–59.6)	97.9 (94.1–99.6)	64.9
Model 6	95.1 (83.5–99.4)	70.6 (63.5–77.1)	41.9 (31.8–52.6)	98.5 (94.6–99.8)	58.7
Model 7	92.7 (80.1–98.5)	79.3 (72.8–84.9)	50.0 (38.3–61.7)	97.9 (94.2–99.6)	66.2
Model 8	85.4 (70.8–94.4)	82.1 (75.8–87.3)	51.5 (39.0–63.8)	96.2 (91.8–98.6)	69.8

*PPV: positive predictive value; NPV: negative predictive value; WR: workload reduction; 95%CI: 95% confidence interval; NLP: natural language processing.*

*Model 1: Original semi-automated algorithm with structured data only.*

*Model 2: Model 1 augmented with NLP component using decision tree and raw counts.*

*Model 3: model 1 augmented with NLP component using decision tree and discretized counts.*

*Model 4: model 1 augmented with NLP component using decision tree and binary counts.*

*Model 5: model 1 augmented with NLP component using random forest and raw counts.*

*Model 6: model 1 augmented with NLP component using random forest and discretized counts.*

*Model 7: model 1 augmented with NLP component using random forest and binary counts.*

*Model 8: Model 1 augmented with a rule-based component*.

## Discussion

In this study, when adding an NLP-component to the original semi-automated algorithm, the number of records to assess manually was decreased by 1.4–12.5% at the cost of sensitivity. The NLP component with the best performance yielded seven (3.6%) fewer patients to review manually, thereby lowering the sensitivity with 2.5% (1 extra deep SSI missed).

Adding the NLP component lowered the number of false positives and thus resulted in WR, however the added value was limited. These findings are similar to a study of Grundmeier et al. [32], who used a data-driven selection of pre-specified keywords related to SSI from clinical narratives after ambulatory paediatric surgery. By using regular expression matching, keyword occurrence was counted and combined within an RF model. High sensitivity (90%) was obtained, but the PPV was 23%, which is lower compared to 44.3–51.5% for the models in our study. A study who successfully succeeded to discriminate between SSI groups is from Thirukumaran and colleagues [21]. They demonstrated high sensitivity and PPV in a model that combined administrative data (age, sex, race, clinical comorbidities, year of procedure and Clinical Classification diagnosis categories) with clinical notes to detect SSIs after orthopaedic surgery. Although they applied a comparable NLP technique as this current study, it remains uncertain what the value of NLP was in case similar structured clinical care data was used as in this current study. Thereby, SSI diagnostic classification after abdominal surgeries is more complex compared to the more ‘straightforward’ orthopaedic surgeries.

Other attempts of NLP surveillance systems from Tvardik et al. [33], Fitzhenry et al. [34], Branch-Elliman et al. [22] and Murff et al. [35] had modest performance results with sensitivities reported between 33% and 87%. All these studies used different NLP techniques, different patient populations and had various targets (other post-operative complications or catheter-related urinary tract infections) complicating direct comparisons, and reflects the numerous techniques available that can be applied to process unstructured clinical notes and to build an algorithm.

The keyword list for the NLP was compiled by various clinical experts from both the Netherlands and Sweden and the NLP component was developed by computer science experts according to cutting edge knowledge and techniques. Despite all this, the addition of the NLP component to the existing algorithm led to minor improvement. This indicates that using clinical notes and NLP for the automated surveillance of SSI after colorectal surgery is not as straightforward as one might expect, and this should be taken into account when designing automated surveillance algorithms. There may be several reasons for the limited benefit obtained in this study by adding an NLP component. First, the NLP component tries to find the patients with deep SSI in an already pre-selected high-risk group identified by the four components of the original algorithm. These patients have either re-operations, antibiotics, radiology or prolonged hospital stays, and certain keywords are therefore expected in all these patients given their clinical course and complications. The lexicon list was probably more focused on distinguishing deep SSI from non-deep SSI, however, maybe other keywords and language patterns are required to identify the deep SSI cases in the high-probability group. Second, on practical ground, we have chosen to add the NLP component as the last step in the algorithm. Including the NLP component in the first steps of the algorithm, as often seen in rule-based algorithms that combine structured and unstructured data, may achieve better results [26]. Third, all studies mentioned above used different techniques to process and analyse clinical notes. We did not attempt all possible options because we investigated NLP in a semi-automation setting, thereby prioritizing sensitivity. Using clinical notes may be more valuable in fully-automated surveillance, in which sensitivity and specificity are balanced instead of focusing on high sensitivity only. However, expectations are tempered as the studies applying other techniques also had modest results. Last, the development of an (NLP) algorithm requires an excellent reference standard of sufficient size to ensure correct classification of patients [10, 11, 22]. Although the agreement between our raters was good, the sample size for developing the NLP components might have been too small.

Clinical notes are a rich data source, useful for post-discharge surveillance and an extremely important data source in the detection and manual ascertainment of SSI by ICPs [36]. It is therefore a logical step to incorporate this data source in surveillance algorithms. However, aside from the limited incremental benefit in this study, several drawbacks of using this data source for automated surveillance exist. First, medical personnel often describe terms indirectly related to SSI (e.g., *dehiscence, opening incision, removing sutures, rupture*) or describe their observations in terms of smell, colour, or shape (e.g., *yellow substance, smelly, not flexible*) making it difficult to catch important vocabulary. Lexicon libraries with medical synonyms, such as the Unified Medical Language System from the National Library of Medicine, can help to connect alternate names for the same concept or keywords, however are not available for all languages (yet) [37]. Second, the frequency of reporting and the vocabulary used varies between individual practitioners, centres and between countries. There is no information available about the generalizability of such algorithms when applied to other languages, and little is known about their robustness – especially when using the count input type – against (local) reporting habits. Third, to the best of our knowledge, there is limited experience with using NLP-augmented surveillance algorithms in daily routines. Given the small benefit that NLP provides in this study, one may wonder whether its development, implementation and maintenance will be cost-effective, at least for deep SSI classification in patients undergoing colorectal surgery. Yet, we have previously shown that using free-text analyses improves surveillance accuracy for urinary tract infections [26]. Although the digital infrastructure can be expanded to other (post-operative) complications, developing and building NLP models require substantial effort of information technology experts. Last, techniques to build NLP-augmented algorithms are mostly complex and less transparent, lowering the chance of understanding and acceptance of clinicians and hospital staff. We used two methods for feature classification. A DT has the benefit of being interpretable, since the tree can be understood as a set of rules for classifying future patients as belonging to either class. A RF, on the other hand, is more complex and therefore lacks in interpretability, but such classifiers are usually more accurate and less likely to over fit data compared to a DT [30]. For future implementation, there will be a trade-off between optimal case-finding techniques versus practical considerations such as acceptability and resources.

## Conclusions

Our study indicated that adding an NLP component to incorporate clinical notes as extra data source lowered the number of false positives, however the benefit was minor as the number of records to review manually was reduced by only 1.4–12.5%. Given the complexity of such systems and the resource-intensive nature of developing NLP, large-scale implementation seems unlikely. However, further research is needed to evaluate whether NLP technology is an appropriate tool for helping to detect deep SSI in semi-automated surveillance systems or their utility in fully-automated surveillance.

## Data Availability

The datasets generated and/or analysed during the current study are not publicly available due to ethical limitations related to sharing patient information, but are available from the corresponding author on reasonable request.
